# HS-SPME Combined with GC-MS/O to Analyze the Flavor of Strong Aroma Baijiu *Daqu*

**DOI:** 10.3390/foods11010116

**Published:** 2022-01-03

**Authors:** Zhe Wang, Song Wang, Pengfei Liao, Lu Chen, Jinyuan Sun, Baoguo Sun, Dongrui Zhao, Bowen Wang, Hehe Li

**Affiliations:** 1 Key Laboratory of Brewing Molecular Engineering of China Light Industry, Beijing Technology and Business University, Beijing 100048, China; 1930201035@st.btbu.edu.cn (Z.W.); wangs_2278@126.com (S.W.); liaopfxs@163.com (P.L.); chenlu_btbu@163.com (L.C.); sunbg@btbu.edu.cn (B.S.); zdr@btbu.edu.cn (D.Z.); wangbw@btbu.edu.cn (B.W.); lihehe@btbu.edu.cn (H.L.); 2 Beijing Laboratory for Food Quality and Safety, Beijing Technology and Business University, Beijing 100048, China

**Keywords:** baijiu, *Daqu*, MHS-SPME, DI-GC-O, volatile flavor components

## Abstract

*Daqu* has gained wide attention because it is an essential source of microorganisms and flavor in baijiu production. In this study, HS-SPME combined with GC-MS/O was used to analyze the volatile flavor components of Strong aroma baijiu *Daqu*. DI-GC-O was used to choose the best extraction fiber to extract the representative overall aroma profile of *Daqu*. A total of 139 compounds were identified in the six different maturity stages of *Daqu*, and these compounds are of different types and concentrations. HS-SPME combined with GC-MS/O was used to analyze the aroma active substances in the finished *Daqu*, and a total of 43 aroma compounds were identified. The OAVs of 21 aromatic compounds were calculated based on the quantitative analysis results of MHS-SPME. Eighteen compounds with OAVs ≥ 1 made significant contributions to the overall aroma of *Daqu*, including guaiacol, 4-ethyl-2-methoxy phenol, 2-ethyl-3,5-dimethylpyrazine, etc.

## 1. Introduction

**Figure 1:**
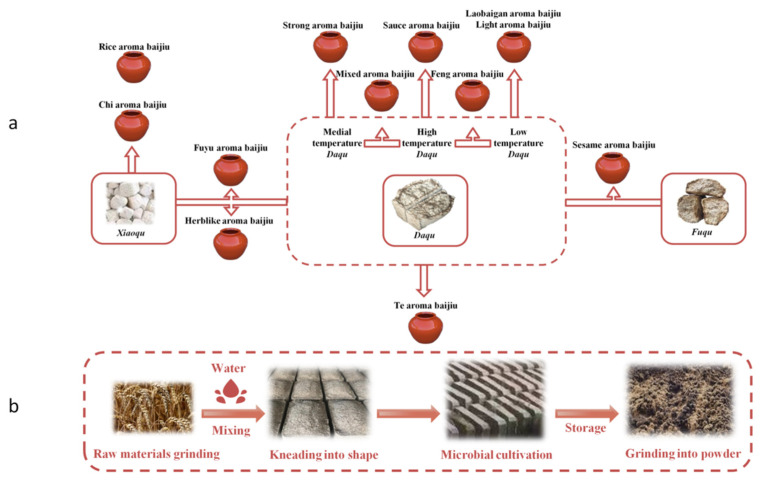
Relationship between 12 flavors of baijiu and the types of *Qu* (**a**), the primary process of *Daqu*-making (**b**).

Baijiu, known as Chinese national liquor, is a part of Chinese traditional culture. As one of the daily alcoholic beverages, it is prevalent among Chinese people [1]. *Qu*, as the source of flavor and microorganism for baijiu, has received wide attention in baijiu production [2,3]. *Qu* is a starter, saccharifying agent and aroma generator in baijiu production, and plays a vital role in baijiu making [1]. The main types of *Qu* are *Daqu*, *Xiaoqu*, and *Fuqu*, and the primary raw materials of *Qu* are wheat, sorghum, and bran, respectively. The relevant microbes that play a major role in the *Qu* fermentation including moulds, yeasts, and bacteria [2]. There are 12 flavor types of baijiu, among which Strong, Light, Sauce, and Rice aroma baijiu are four basic flavor types, and other flavor types are derived from the basic flavors [4]. The 12 flavor types of baijiu are closely related to the kinds of *Qu*. According to the main technical characteristics of the 12 flavor types of baijiu and the primary production technology of *Qu*, the relationship between flavor types and *Qu* is summarized as shown in Figure 1a.

Flavor wheel has been often used to help people understand and identify the flavor of a complicated product. The Baijiu flavor wheel [5] describes the sensory characteristics of baijiu from three aspects: aroma, taste, and mouthfeel (Figure S1). The aroma of raw materials, fermentation aroma, and aging aroma are three important aroma components for the Baijiu Flavor Wheel. *Qu* aroma is one of the seven kinds of aroma to evaluate raw materials aroma. *Daqu* used in SAB is a kind of *Qu*. Crushed wheat and other raw materials are mixed with an appropriate amount of water to produce *Daqu*, which is then made into *Qu*-bricks with a specific mold. Then, the *Qu*-bricks were transferred to the fermentation room, appropriate straw use for isolation and moisture absorption. Pile the fermented *Qu*-bricks in the store room to be crushed for use. The immediate process of *Daqu*-making is demonstrated in Figure 1b. *Daqu* contains high protein and is rich in starch and other nutrients, which is the primary source of microorganisms [3]. It can provide microorganisms with hydrolytic enzymes that hydrolyze macromolecules in fermented grains. In the process, an aroma is produced, which we call *Qu* aroma. Strong aroma baijiu (SAB) is one of the baijiu flavor types accounting for more than 70% of the market, for which sorghum, rice, sticky rice, wheat, and corn are used as the raw material. Its standard production technology is steamed grains mixed with *Daqu* are put into *pit* (*jiaochi*) for fermentation. After fermentation in the pit, solid-state distillation occurs with *zeng* (a kind of distillation device). Containers are used to collect and store baijiu. At present, the research on the flavor of SAB is aimed at the study of the overall flavor of baijiu [6], the change rule of flavor substances in the baijiu distillation process [7] and the characteristic aroma: roasted and mud-like aromas [8]. This study is carried out in view of the problem that the research on *Qu* aroma is not sufficient.

The samples in this experiment are six kinds of *Daqu* from the freshly pressed *Daqu* block in the production process to the *Daqu* before it is officially put into SAB production. At present, there are mainly two methods for the extraction of volatile compounds from solid samples: (1) To make the volatile compounds of the samples reach equilibrium in the headspace in the confined space, and then use the extraction fiber coated with unique materials to extract directly from the headspace gas of the samples; (2) To extract the volatile compounds from the solid to the liquid by using the principle of compound similarity compatibility. Then, the liquid sample was concentrated and extracted by the pretreatment method. At present, the pretreatment methods applied to the analysis of volatile components in solid samples are mainly solid phase microextraction (SPME), simultaneous distillation extraction (SDE), solvent assisted flavor evaporation (SAFE), etc. In this paper, HS-SPME was used to extract the aroma of *Daqu*.

**Figure 2:**
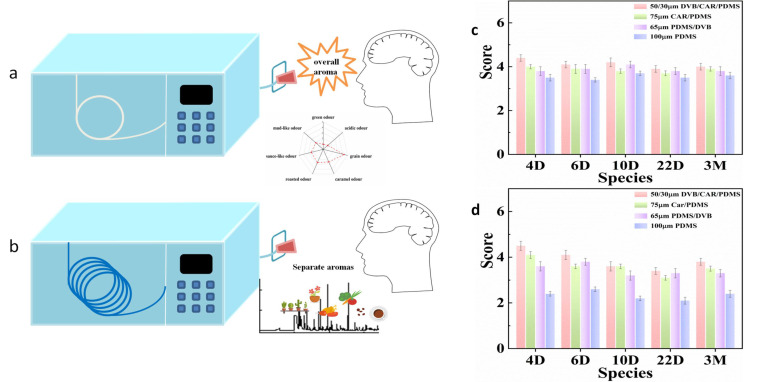
DI-GC-O sniffing diagram (**a**), with the short unfilled capillary column. GC-O sniffing diagram (**b**), with the packed column. Screening and optimization of extraction head by DI-GC-O Aroma similarity (**c**) and aroma intensity (**d**).

Headspace solid phase microextraction (HS-SPME) is an incomplete method, which is easily affected by the matrix effect in the quantitative analysis of compounds in solid samples, which leads to inaccurate quantification. Multiple headspace solid phase microextraction (MHS-SPME) is a quantitative method developed based on HS-SPME. MHS-SPME has nothing to do with the distribution between the two phases, which can eliminate the matrix effect of the sample. At present, MHS-SPME has been successfully applied to the quantification of some volatile compounds in mushrooms [9,10], tomatoes [11], cheese [12], dry fermented sausage [13], wine [14,15] and alcoholic beverages [16]. This paper is the initial stage of the application of MHS-SPME to the analysis of volatiles in *Daqu* as we know it. Direct gas chromatography olfactometry (DI-GC-O) is a direct and effective technique to evaluate sensory flavor [17]. The extract was injected into a short unfilled capillary column directly connected to the GC sniffing port. Since there was no chromatographic separation, the method could evaluate the overall olfaction of the extract [18]. As shown in Figure 2, the volatile odors not separated by the chromatographic column (a) are smelled more quickly and directly than the column with packing (b). It has been used in the detection of orange juice [17], soy sauce [18], and baijiu samples [19,20] at present. Many researchers select extraction fiber according to the peak area of compounds. However, some studies showed that the aroma intensity detected by DI-GC-O correlated with the total peak area analyzed by GC-MS [18]. Therefore, DI-GC-O is listed as a good choice. The application of this method in solid samples such as *Daqu* has not been reported.

This research will introduce from the following aspects: (1) the extraction fiber was chosen with DI-GC-O; (2) analyses of aroma-active compounds from different maturity stages *Daqu* by HS-SPME combined with GC-MS/O; (3) key odor compounds of *Daqu* based on MHS-SPME quantitative analysis and aroma active value (OAV) calculation; and (4) it provides scientific support for the use of the stored *Daqu* in actual production in terms of its aroma richness.

## 2. Materials and Methods

### 2.1. Chemical Standards, Reagents, and Materials

2,6-dimethylpyrazine, nonanal, (*E*)-2-octenal, 2,3-dimethylpyrazine, 3-ethyl-2-methylpyrazine, 2,3,5-trimethylpyrazine, 2-ethyl-3,5-dimethylpyrazine, benzaldehyde, (*E*)-2-nonanal, *β*-caryophyllene, *γ*-butyrolactone, phenylacetaldehyde, furfuryl alcohol, 2,4-decadienal, geranyl acetone, guaiacol, 4-ethyl-2-methoxyphenol, *γ*-nonanolactone, 2-methoxy-4-vinylphenol, hexanal, 2-phenyl-2-butenal (all of them are chromatographic purity with at least 97% purity, J&K Scientific Ltd.). A C7−C30 n-alkane mixture was purchased from J&K Scientific Ltd., Beijing, China. The internal standard 2-octanol (chromatographic purity with 98.0% purity), anhydrous ethanol (chromatography grade), and sodium chloride (analytical purity) were purchased from China National Pharmaceutical Group Corp. (Shanghai, China). High purity helium (99.999%) was purchased from Beijing Yanglilai Chemical Gas Co., Ltd. (Beijing, China).

### 2.2. Samples

All were collected from the Gujing gongjiu Co., Ltd., Anhui, China. All samples were stored at −20 °C separately in sealed plastic bags in the fridge until analysis. 

There are several key time points for the fermentation process of the *Daqu* that we distinguish by the number of days. The samples for this experiment were collected for these key time points. The information of the sample is shown in Table S1.

### 2.3. Sample Pretreatment

The *Daqu* samples were smashed and screened over 40 mesh. Each different maturity stage of *Daqu* (as shown in Table S1) is mixed individually—sealed and stored in cold storage to ensure the reproducibility of sample extraction.

### 2.4. Extraction of Volatile Components of Daqu

#### 2.4.1. Selection of Extraction Fiber by DI-GC-O

In this experiment, *Daqu* S-3M was used. The experimental tasters were composed of three highly trained tasters in our research group (All of them are members of the Key Laboratory of Brewing Molecular Engineering of China Light Industry who have received sensory training of baijiu evaluation). 

In addition, 1.5 g *Daqu* sample was taken and placed in a cup at room temperature of 25 °C ± 1 as reference. The overall odor intensity of *Daqu* S-3M was scored directly. The intensity of aroma was rated on 6-point scale. 0 = not smell, 1 = very weak smell, 2 = weak smell, 3 = moderate smell, 4 = strong smell, and 5 = very strong smell, and the overall flavor of samples was memorized.

Under the condition of different extraction fibers, the aroma components analyzed by DI-GC-O but not separated by a chromatography column were scored according to the flavor intensity of real *Daqu*. In the experiment process, the members of the sniffing group need to evaluate the samples to be tested in two aspects: (1) the overall flavor intensity of olfactory perception; (2) between the flavor of the extract and the original sample similarly. The reviewers need to sniff the actual sample again between the two sniffing experiments to enhance their impression. Each person sniffed three times and scored, averaging the results based on each person’s score. 

Gas chromatographic conditions: no-packed capillary column (100 cm × 25 mm). The front inlet temperature was 250 °C in splitless mode. The extraction fiber was inserted into the injection port of a GC for 5 min to desorb, N_2_ (99.999%) as the carrier gas at 2.0 mL/min. The oven was 40 °C initially. Between the two injections, the column temperature was raised to 250 °C for 5 min, and the column was aged to prevent cross contamination.

#### 2.4.2. Volatile Components Extracted by HS-SPME

The extraction fiber was screened according to the previous experiment, the 50/30 μm DVB/CAR/PDMS fiber was selected as extraction fiber, and 50 °C as extraction temperature and 40 min as extraction time. In addition, 5 g of different maturity stages *Daqu* samples were weighed in a 20 mL headspace bottle, equilibrated at 50 °C for 20 min and extracted for 40 min. Then, the extraction fiber was placed at 250 °C for 5 min resolution time in the GC injection port, analyzed with GC-MS subsequently.

The heating procedure, GC, and Mass spectrum conditions were using conditions in reference [21].

### 2.5. Identification of Volatile Components

#### 2.5.1. Volatiles Identification by GC-MS/O Analysis

During the analysis, the evaluator must place the nose at the olfactory detector and record the retention time, aroma characteristics, and odor intensity during the olfactory process. Among them, the intensity range is 1–4, and 1 means weak aroma intensity; 2 means the aroma intensity is slightly stronger; 3 means obvious aroma intensity; 4 means strong aroma intensity. Each person sniffed three times to ensure the accuracy of the results.

GC conditions: DB-WAX column (60 m × 0.25 mm × 0.25 μm) and HP-5 column (30 m × 0.25 mm × 0.25 μm); Helium (99.999%) was used as a carrier gas at a constant flow rate of 1.0 mL/min. The temperature of the injection port was 250 °C, and the resolution time was 5 min.

The heating procedure was the same as the Section 2.4.2 heating procedure.

Olfactory detector: With the carrier gas, the effluent from the column is divided into the mass spectrometer detector and the olfactory detector in 1:1 mode. The olfactory temperature and the transmission line temperature of the olfactory end are 250 °C. To prevent the nose from drying out after a long time of sniffing, the olfactory temperature and nitrogen line are passed through deionized water.

#### 2.5.2. Qualitative Analysis

NIST 11 library search, standard comparison, and retention index were used for qualitative analysis. 

NIST 11 library search: gas chromatography-mass spectrometry data are analyzed and processed by Chemstation software (1989–2010 Agilent Technologies, Inc., Santa Clara, CA, USA). Firstly, the background of the spectrum was deducted, and then the library search was carried out. The qualitative results of the library were required to have a similarity of more than 80%.

Standard comparison: under the same chromatographic conditions, the sample and standard can be analyzed. If the retention time and mass spectrum of the corresponding peaks of the two chromatograms are the same, they can be identified as the same compound.

Retention index (RI) qualitative was calculated [6] using a C7~C30 n-alkane mixture under the same GC-MS conditions as the samples.

### 2.6. Quantitative and OAV Analysis

MHS-SPME is used as a quantitative method. The appropriate sample volume is the critical factor in obtaining the exponential decrease of peak area of each analyte in the continuous extraction process. In this experiment, the previous experimental methods and results [21] were used, for which 35 mg samples were selected for analysis. Weigh 35 mg of *Daqu* sample into a 20 mL headspace bottle, and carry out the experimental operation according to the HS-SPME method described before. The interval between two adjacent extractions was 20 min.

Quantitative: calibration with an external standard curve, take 100 μL diluted the mixed standard solution in the headspace bottle, and then analyze by MHS-SPME under the same conditions as the sample. All analyses were repeated in triplicate.

The OAV value was calculated based on the ratio of the target odorant concentration to its threshold.

### 2.7. Statistical Analysis

The compounds were analyzed by Chemical Station (Agilent, Santa Clara, CA, USA). Origin Pro 2021 was used for data analysis and related graphics drawing. SIMCA-14.1 was used to analyze PCA (Umetrics, Umea, Sweden).

## 3. Results

### 3.1. Screening and Optimization of Extraction Fiber by DI-GC-O

This method injected extraction fiber with an adsorbed compound into an empty chromatographic column connected with the GC injection port and olfactory detector. Then, the sensory evaluator directly smelled the aroma extract which was not separated by the chromatographic column. The best extraction conditions determined by the overall flavor similarity combined with sensory evaluation between the extract and the real sample were investigated. This method avoids the separation effect of the chromatographic column and restores the authenticity of the sample to the greatest extent. DI-GC-O was used to evaluate the extraction effect of four different extraction fibers in *Daqu*. 

The order of overall aroma intensity and similarity of different extraction fibers is 50/30 μm DVB/CAR/PDMS > 75 μm CAR/PDMS > 65 μm PDMS/DVB > 100 μm PDMS, which can be seen from Figure 2c,d. It shows that the 50/30 μm DVB/CAR/PDMS extraction fiber has certain advantages in the extraction of aroma compounds in *Daqu*, which can extract more compounds and most likely reflect the overall aroma of *Daqu*. 

### 3.2. Analysis of Daqu at Different Maturity Stages by HS-SPME Combined with GC-MS

**Figure 3:**
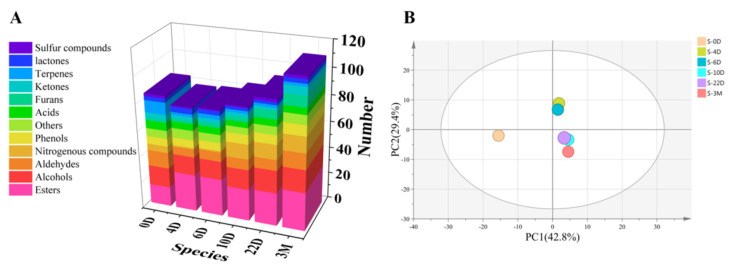
Types and changes of volatile components in *Daqu* at different maturity stages (**A**); PCA score plots of different maturity stages *Daqu* (**B**).

According to the HS-SPME extraction method in Section 2.4.2, 139 compounds were identified in six different maturity stages *Daqu* samples (shown in Table 1), including 36 esters, 7 acids and ketones, 22 alcohols, 4 lactones, 8 phenols and furans, 14 aldehydes, 12 nitrogenous compounds, 9 terpenes, 3 sulfur compounds and 9 others. The number of volatile compounds in six different mature stages *Daqu* was in the order of “S-3M > S-22D > S-10D = S-0D > S-6D > S-4D”. The variation of compounds types in *Daqu* is shown in Figure 3A, and it can be found that the aroma of *Daqu* S-3M was more abundant.

Esters, which account for 60% of all flavor substances in baijiu, play an essential role in baijiu [22]. It can be seen from Table 1 and Figure 3A that the ester compounds in *Daqu* (S-4D) after early fermentation increased significantly, and the relative peak area reached 43.381%. With the fermentation, the relative peak areas of esters in S-10D, S-22D and S-3M were more than 50%, the kinds of esters increased, and the content of long-chain fatty acid esters such as ethyl pentadecanoate, ethyl octadecenoate, and ethyl hexadecanoate increased significantly.

The relative content of nitrogen-containing substances also increased significantly with the extension of fermentation time. After storage, the range of nitrogen-containing substances in *Daqu* reached 7.228%. Nitrogen-containing substances significantly increased after a slow rising period (about S-10D) compared with ester compounds. Among them, the content of tetramethylpyrazine changed the most, which was the most nitrogen-containing compound in *Daqu* after storage. This is because nitrogen-containing substances were mainly produced by the Maillard reaction. With the fermentation, the fermentation temperature of medium high temperature *Daqu* could reach 50–60 °C. In the high temperature transformation stage of no less than one week, amino acids and proteins in wheat undergo the Maillard reaction to produce a variety of pyrazines (one of the nitrogenous compounds) [23]. The compounds mainly show a roasting and nut-like aroma, which might contribute to the flavor of the finished *Daqu*. Nitrogen-containing substances in *Daqu* might be brought into baijiu.

The number of aldehydes and ketones decreased first and then increased. *Daqu* used in production is made from wheat, rich in aldehydes and ketones [24]. Therefore, the type and content of aldehydes and ketones in S-0D are relatively high, which might be related to the high content of aldehydes and ketones in grain raw materials. Ten species of aldehydes decreased significantly after the early fermentation (about S-4D) from Table 1. With the progress of fermentation, aldehydes and ketones were generated by non-enzymatic chemical reactions. 

Acid compounds are the primary metabolites and the premise substances for the synthesis of other compounds. The changes in the fermentation process are mainly reflected in the changes in the relative content. After the early fermentation (about S-4D), the range of acid compounds reached the maximum (11.086%) and then decreased slowly. Alcohols and phenols are primary metabolites, showing the same trend of change. The content of alcohols and phenols first increased and then also reduced during *Daqu* ripening.

Furan compounds have furan heterocyclic structure and strong activity. With the fermentation, the kinds of furan compounds increased significantly, but the overall content of furan compounds decreased. Eight furan compounds were identified in *Daqu* after storage (S-3M). Furfuryl alcohol content in S-3M increased, which showed coffee aroma, malt aroma, and baking aroma.

Terpenes with isoprene as a structural unit multiple and their oxygen-containing derivatives widely exist in plants [25]. Therefore, the species (9 species) and content (27.399%) of *Daqu* (S-0D) were higher, and the species and content decreased significantly after early fermentation.

PCA analysis was performed based on data obtained from area percentage at different maturity stages *Daqu* in Table 1 to reveal the distribution of samples and detect outliers. PCA score plots of varying maturity stages *Daqu* were shown in Figure 3B. The first principal component (PC1) accounted for 42.8% of the variance, and the second principal component (PC2) accounted for 29.4%. Two components (PC1 and PC2) explained 72.2% of the variance. Different maturity stages *Daqu* were separated in the PCA score plots. The variables have no outliers and are within the 95% confidence interval. According to Figure 3B, the S-0D sample was significantly different from the others, and the S-4D and S-6D fermentation samples showed similarities. In addition, S-10D, S-22D, and S-3M have a certain similarity, but S-3M can be appropriately distinguished from the other two samples.

### 3.3. Analysis of Active Aroma Compounds in Daqu S-3M by GC-MS/O

According to the qualitative results in Table 1, the volatile components of *Daqu* at different maturity stages are quite different. The greater variety and content of aromas in the *Daqu* S-3M determined the final use of it in actual production. The following *Daqu* S-3M was taken as the object to explore the active aroma compounds. 

GC-MS/O analyzed the active aroma compounds in *Daqu* S-3M. A total of 50 aroma regions were smelled under different polarity chromatographic columns, except for seven compounds that were not successfully identified. Forty-three compounds were identified by mass spectrometry, retention index, and standard substance as shown in Table 2: including 8 esters, 2 lactones, 7 alcohols, 8 aldehydes, 5 nitrogenous compounds, 4 phenols and acids, 3 terpenes, 1 sulfur compound, and another compound (naphthalene).

In Table 2, No. 6 (2,6-dimethylpyrazine), No. 8 (dimethyl trisulfide), No. 14 (acetic acid), No. 16 (2-ethyl-3,5-dimethylpyrazine), No. 19 (benzaldehyde) No. 22 (*β*-caryophyllene), No. 24 (butanoic acid), No. 29 (3-methylbutanoic acid), No. 41 (guaiacol), No. 43 (phenethyl alcohol), No. 45 (4-ethyl-2-methoxyphenol), No. 46 (*γ*-nonanolactone), and No. 49 (methyl palmitate) had higher olfactory intensity. In addition, many compounds with low olfactory intensity might affect the overall aroma profile of *Daqu* [26] because they can have high flavor thresholds.

Esters are the main flavor substances in baijiu, and most of the short-chain esters with the flavor of fruit odor, including No. 2 (ethyl butyrate), No. 3 (ethyl valerate), and No. 5 (ethyl hexanoate). Long-chain fatty acid esters, such as methyl palmitate, have a high threshold but show a significant wax flavor, which may be related to the characteristic aroma of *Daqu* [23]. There are many types of esters, and their olfactory intensities are relatively low except for No. 49 (methyl palmitate). In addition, among the three lactones identified in S-3M, *Daqu*, *γ*-nonanolactone, and *γ*-butyrolactone showed aroma activity, and *γ*-nonanolactone with coconut flavor also had high aroma intensity. The results showed that lactones played a significant role in the aroma of baijiu, while *γ*-nonanolactone was one of the essential aroma compounds.

A large number of alcohols were identified in this experiment, but the olfactory intensity was low. The smell intensity of phenethyl alcohol and benzyl alcohol containing a benzene ring was relatively high, showing flower, sweet, and honey fragrance.

In addition to No. 44 (2-phenyl-2-butenal, sweet aroma), *Daqu* also had more aldehydes after storage, which mainly showed a typical compound aroma of grass and oil, bitter almond, and so on. It can be seen from Table 2 that the relative content of No. 44 (2-phenyl-2-butenal) among the seven aldehydes identified in *Daqu* was S-0D (0.037%) < S-3M (0.457%). In addition, the relative content of nonanal, (*E*)-2-octenal, phenylacetaldehyde, (*E*)-2-nonenal, phenylacetaldehyde, and 2,4-decadienal is S-0D > S-3M. The compound aroma of these aldehydes has an essential influence on the flavor of *Daqu*.

Phenol compounds have high odor intensity, which were important aroma active compounds in *Daqu*. Guaiacol, 4-ethyl-2-methoxyphenol, and 2-methoxy-4-vinylphenol were characterized with wood flavor, clove, scorch bitter taste, etc., while *p*-cresol has an animal odor.

Pyrazine compounds (nitrogenous compounds) are considered the primary source of coke flavor and baking flavor in *Daqu* [23]. In addition, 2,5-dimethylpyrazine and 2,6-dimethylpyrazine are considered essential aroma compounds in medium and high temperature *Daqu*. The five pyrazines identified in this experiment are wheat flavor, roasted flavor, and potato flavor.

Dimethyl trisulfide (garlic) and naphthalene (grass) also had higher olfactory intensity in *Daqu*. Dimethyl trisulfide was considered a vital aroma substance in SAB [6]. According to the above speculation, *Daqu* and baijiu have common flavor compounds, and they are related on flavor.

### 3.4. Quantitative Analysis of Aroma Components in Daqu by MHS-SPME

The quantitative results of 21 compounds in *Daqu* have been reported in a previous article [21]. Quantitative Results showed the data of the standard curve obtained by MHS-SPME: the correlation coefficient (R^2^) is between 0.9717 and 0.9983, and the fitting is good. The LOD of all compounds was less than 68.4 ppb, which showed high sensitivity. The RSD of the compounds was calculated using the results of three replicates. The RSD of each compound was lower than 11.75%. The experimental repeatability was good at the same time. The recoveries of the compounds ranged from 83.58% to 120.05%, which met the needs of experimental quantification. In the quantitative analysis, the content range was 12.93 ng/g-2816.28 ng/g. The content of furfuryl alcohol (2816.28 ng/g) with the burned taste was the highest. In addition, the content of phenylacetaldehyde, guaiacol, nonanal, and *γ*-butyrolactone was higher than 500 ng/g. The contents of 2-phenyl-2-butenal, 2-ethyl-3,5-dimethylpyrazine, geranyl acetone, and 3-ethyl-2-methylpyrazine were lower than 100 ng/g.

The content of the compound only indicates the amount of the compound in the sample, which can not reflect the effect of the compound on the overall flavor of the sample. For example, furfuryl alcohol had the highest content, but its olfactory intensity was low. Therefore, based on quantitative analysis, combined with the threshold value of compounds, OAV value was calculated to evaluate the effect of different compounds on the overall flavor of samples [26].

The OAV values of 21 compounds were calculated as shown in Table 3, and 18 compounds OAVs ≥ 1, which contributed greatly to the overall aroma of *Daqu* and were important flavor compounds in *Daqu* aroma. In exploring Baijiu flavor, grain aroma is often described as one of the flavor descriptors and is an important flavor branch of baijiu flavor quality evaluation. Nine compounds related to “grain aroma” have higher OAV values except for geranyl acetone (OAV = 1). (*E,E*)-2,4-decadienal has the highest OAV value (OAV = 2746) among them. (*E,E*)-2,4-decadienal (OAV = 2746), nonanal (OAV = 655), phenylacetaldehyde (OAV = 447), (*E*)-2-nonenal (OAV = 339), hexanal (OAV = 81), 4-ethyl-2-methoxyphenol (OAV = 40), (*E*)-2-octenal (OAV = 40), and *γ*-nonanolactone (OAV = 10) have an important contribution to *Daqu* aroma. It was consistent with other literature that nonanal and 4-ethyl-2-methoxyphenol were considered aroma active substances in *Daqu* [26].

In addition, eight compounds had OAV ≥ 1. Among them, guaiacol (OAV = 172), 2-methoxy-4-vinylphenol (OAV = 122), 2-ethyl-3,5-dimethylpyrazine (OAV = 43), furfuryl alcohol (OAV = 28), 2,3,5-trimethylpyrazine (OAV = 24), 2-ethyl-3,5-dimethylpyrazine (OAV = 22), and *γ*-butyrolactone (OAV = 11) were higher.

## 4. Conclusions

Under the optimal extraction conditions, 139 compounds are identified in the six different maturity stages of *Daqu*. Through the analysis of the variety of compounds and the PCA analysis of the compound contents, the scientific basis for *Daqu* S-3M widely used in practical production is provided from the flavor perspective. In the maturing process of *Daqu*, ester compounds were mainly produced in the early stage of fermentation, and pyrazine compounds were mainly produced after the slow rising stage, and the content and species were relatively stable after that. The number of aldehydes and ketones decreased first and then increased. The content of acids reached the maximum after early fermentation and then decreased slowly. The contents of alcohols and phenols increased at first and then decreased during *Daqu* ripening. The kinds of furan compounds increased with the fermentation. The types and contents of terpenoids decreased obviously after early fermentation.

In this experiment, HS-SPME combined with GC-MS/O was used to analyze the active aroma substances in the *Daqu* S-3M, and 42 active aroma compounds were identified. Among the 21 aroma compounds quantitatively analyzed, 18 compounds with OAV ≥ 1 were important in *Daqu* aroma including guaiacol, 4-ethyl-2-methoxy phenol, 2-ethyl-3,5-dimethylpyrazine, furfuryl alcohol, 2,3,5-trimethylpyrazine, etc. It provides a reference for the study of *Daqu* from the perspective of flavor. This provides a scientific basis for the use of *Daqu* in practice.

## Figures and Tables

**Table 1:** Analysis of volatile components in *Daqu* at different maturity stages.

No.	Compound	CAS Number	RI	Identification ^a^	Area Percentage % ^b^					
					S-0D	S-4D	S-6D	S-10D	S-22D	S-3M
Esters										
1	Ethyl 3-methylbutanoate	108-64-5	1074	MS,RI	—	0.525	0.765	—	—	—
2	Isoamyl acetate	123-92-2	1123	S,MS,RI	—	1.126	0.159	—	—	0.082
3	Ethyl pentanoate	539-82-2	1135	S,MS,RI	—	—	—	—	0.335	—
4	Ethyl hexanoate	123-66-0	1233	S,MS,RI	3.760	2.489	1.230	8.698	8.656	1.122
5	Ethyl heptanoate	106-30-9	1334	MS,RI	—	0.367	0.330	0.485	0.505	0.270
6	Ethyl lactate	97-64-3	1344	S,MS,RI	—	0.800	0.330	—	—	0.530
7	Hexyl formate	629-33-4	1350	S,MS,RI	2.136	0.159	0.137	0.869	0.358	0.562
8	1-Methylheptyl acetate	2051-50-5	1363	MS	—	—	—	—	0.038	—
9	Ethyl octanoate	106-32-1	1435	S,MS,RI	1.514	1.263	1.177	0.518	0.796	0.404
10	Ethyl nonanoate	123-29-5	1537	S,MS,RI	—	0.159	0.179	0.587	0.345	0.237
11	2-Hydroxyethyl hexanoate	6946-90-3	1545	MS,RI	—	1.789	—	—	—	—
12	3-(Methylthio)-propanoic acid ethyl ester	13327-56-5	1572	MS,RI	—	0.665	0.268	0.111	0.132	0.137
13	Hexyl hexanoate	6378-65-0	1611	S,MS,RI	—	—	—	—	—	0.066
14	Ethyl 2-furoate	614-99-3	1631	MS,RI	0.230	—	—	—	—	—
15	Ethyl decanoate	110-38-3	1640	S,MS,RI	—	1.056	1.231	0.292	0.343	0.223
16	Ethyl benzoate	93-89-0	1674	S,MS,RI	0.365	0.380	0.331	0.289	0.150	0.129
17	Diethyl succinate	123-25-1	1681	S,MS,RI	0.118	3.024	1.132	0.075	0.490	0.582
18	Methyl salicylate	119-36-8	1786	S,MS,RI	0.290	0.249	0.114	0.382	0.482	0.371
19	Ethyl phenylacetate	101-97-3	1793	S,MS,RI	0.199	3.155	0.445	1.034	0.765	0.815
20	Methyl dodecanoate	111-82-0	1803	S,MS,RI	0.084	0.017	0.026	0.088	0.066	0.280
21	Ethyl 2-hydroxybenzoate	118-61-6	1820	S,MS,RI	—	—	—	—	0.069	0.198
22	Phenethyl acetate	103-45-7	1824	S,MS,RI	—	0.559	0.080	0.154	—	—
23	Ethyl nicotinate	614-18-6	1825	S,MS,RI	0.063	—	0.110	0.136	0.119	—
24	Ethyl dodecanoate	106-33-2	1843	S,MS,RI	—	0.689	0.889	0.562	0.596	0.480
25	Ethyl 3-phenylpropanoate	2021-28-5	1884	S,MS,RI	0.125	0.103	0.082	0.033	0.113	0.052
26	Phenylethyl isovalerate	140-26-1	1978	S,MS	—	0.025	0.021	—	—	0.015
27	Ethyl tetradecanoate	124-06-1	2022	S,MS,RI	0.182	2.003	2.891	2.712	3.127	0.100
28	Ethyl cinnamate	103-36-6	2094	S,MS,RI	—	—	—	—	—	0.042
29	Ethyl pentadecanoate	41114-00-5	2099	S,MS,RI	—	0.494	0.715	1.320	1.186	1.251
30	Methyl hexadecanoate	112-39-0	2181	MS	—	—	—	—	—	0.118
31	Ethyl hexadecanoate	628-97-7	2227	S,MS,RI	2.777	14.552	18.454	23.904	22.731	29.140
32	Ethyl 9-hexadecenoate	54546-22-4	2259	S,MS,RI	—	1.298	1.589	0.882	1.226	0.997
33	Ethyl heptadecanoate	14010-23-2	2350	S,MS,RI	—	0.056	0.067	0.114	—	0.117
34	Ethyl octadecanoate	111-61-5	2483	S,MS,RI	—	0.318	0.364	0.602	0.564	0.648
35	Ethyl Oleate	111-62-6	2475	S,MS,RI	0.457	2.843	3.961	6.784	6.399	7.778
36	Methyl octadecadienoate	112-63-0	2588	S,MS	0.107	3.214	3.614	5.067	6.111	6.372
	Subtotal				12.410	43.381	40.692	55.695	55.703	53.120
Acids										
37	Acetic acid	64-19-7	1458	S,MS,RI	0.756	0.487	0.562	0.674	0.851	0.729
38	2-Methylpropionic acid	79-31-2	1569	MS,RI	—	0.645	0.214	0.077	0.077	0.085
39	3-Methylbutanoic acid	503-74-2	1672	MS,RI	0.251	9.765	3.172	1.224	1.596	1.514
40	Hexanoic acid	142-62-1	1846	S,MS,RI	1.253	0.165	0.037	0.288	0.185	0.336
41	Heptanoic acid	111-14-8	1940	MS,RI	0.101	0.024	0.036	—	—	—
42	Octanoic acid	124-07-2	2031	S,MS,RI	0.241	—	—	—	0.081	0.053
43	Decanoic acid	334-48-5	2247	MS,RI	0.357	—	—	—	—	—
	Subtotal				2.959	11.086	4.021	2.263	2.789	2.717
Alcohols										
44	3-Methyl-1-butanol	123-51-3	1202	MS,RI	0.373	4.540	4.454	2.077	0.514	4.323
45	1-Pentanol	71-41-0	1246	MS	0.827	—	—	—	—	0.873
46	3-Methyl-2-buten-1-ol	556-82-1	1321	S,MS,RI	—	—	—	0.089	0.020	0.342
47	3-Octanol	589-98-0	1391	MS,RI	0.328	—	—	—	0.415	—
48	1-Octen-3-ol	3391-86-4	1448	S,MS,RI	0.854	0.484	0.826	0.271	0.237	0.311
49	1-Heptanol	111-70-6	1452	S,MS,RI	1.342	0.055	0.126	0.671	0.497	0.151
50	6-Methyl-5-hepten-2-ol	1569-60-4	1462	MS,RI	0.489	—	—	—	—	—
51	2-Ethylhexanol	104-76-7	1488	S,MS,RI	0.874	0.040	0.057	0.545	0.193	0.413
52	2-Nonanol	628-99-9	1517	S,MS,RI	—	0.083	0.101	0.180	0.095	0.043
53	1-Octanol	111-87-5	1556	S,MS,RI	0.428	0.053	0.066	0.148	0.097	0.139
54	2,3-Butanediol	513-85-9	1577	S,MS,RI	1.364	0.056	0.083	0.646	0.403	0.699
55	(*E*)-2-Octenol	18409-17-1	1616	S,MS,RI	0.224	0.081	0.112	—	—	—
56	1-Nonanol	143-08-8	1659	S,MS,RI	0.166	0.055	0.051	0.054	0.056	0.061
57	(*Z*)-3-Nonen-1-ol	10340-23-5	1684	MS,RI	0.104	—	—	—	—	—
58	*α*-Terpineol	98-55-5	1699	S,MS,RI	0.747	0.038	0.039	0.042	0.281	0.038
59	Methionol	505-10-2	1721	S,MS,RI	—	0.071	0.118	0.108	0.046	0.047
60	2-Phenyl-2-propanol	617-94-7	1762	S,MS,RI	—	—	—	0.117	0.037	0.097
61	1-Phenylethanol	98-85-1	1819	S,MS,RI	—	—	—	—	0.057	0.019
62	Benzyl alcohol	100-51-6	1878	S,MS,RI	0.188	0.228	0.176	1.318	1.033	2.233
63	Phenylethyl Alcohol	60-12-8	1907	S,MS,RI	3.652	14.526	11.272	7.081	14.432	11.298
64	2-Phenyl-1-propanol	1123-85-9	1923	S,MS,RI	—	—	—	0.052	0.070	0.047
65	2-Phenyl-1-butanol	2035-94-1	1973	MS	—	0.025	0.041	—	—	—
	Subtotal				11.960	20.335	17.523	13.400	18.482	21.134
lactones										
66	*γ*- Butyrolactone	96-48-0	1643	S,MS,RI	0.130	0.029	0.136	0.195	0.164	0.113
67	5-Butyldihydro-2(3H)-furanone	104-50-7	1917	S,MS,RI	0.133	0.156	0.179	—	—	0.106
68	*γ*- Nonanolactone	104-61-0	2017	S,MS,RI	1.445	0.389	0.237	0.142	0.142	0.162
69	(*Z*)-6-Dodeceno-γ-lactone	18679-18-0	2438	S,MS	—	0.076	—	—	—	—
	Subtotal				1.708	0.650	0.552	0.337	0.307	0.382
Phenols										
70	Guaiacol	90-05-1	1865	S,MS,RI	0.177	—	—	1.150	0.136	0.172
71	Creosol	93-51-6	1952	S,MS,RI	—	0.432	0.029	0.101	0.078	0.073
72	*o*-Cresol	95-48-7	1990	S,MS	—	—	—	—	—	0.029
73	Phenol	108-95-2	1993	S,MS,RI	0.400	0.041	0.054	0.874	0.285	0.373
74	4-Ethyl-2-methoxyphenol	2785-89-9	2015	S,MS,RI	3.002	13.076	17.783	0.555	0.943	0.490
75	4-Ethylphenol	123-07-9	2142	S,MS,RI	0.141	0.091	0.112	0.071	0.034	0.137
76	2-Methoxy-4-vinylphenol	7786-61-0	2177	S,MS,RI	0.646	3.237	8.710	0.969	0.696	0.319
77	2,4-Bis(1,1-dimethylethyl)-phenol	96-76-4	2296	S,MS,RI	10.538	0.135	0.291	1.228	0.611	0.962
	Subtotal				14.903	17.012	26.978	4.949	2.785	2.554
Ketones										
78	3-Octanone	106-68-3	1254	S,MS,RI	—	0.114	0.194	—	—	—
79	2-Octanone	111-13-7	1286	S,MS,RI	5.823	4.586	4.793	7.414	8.267	2.411
80	6-Methyl-5-hepten-2-one	110-93-0	1340	S,MS,RI	0.296	—	—	—	—	0.316
81	2-Nonanone	821-55-6	1390	S,MS,RI	—	—	—	0.143	—	0.214
82	3,5-Octadien-2-one	38284-27-4	1525	MS,RI	0.083	—	—	—	—	0.030
83	Acetophenone	98-86-2	1661	S,MS,RI	0.701	0.082	0.104	0.594	0.521	0.871
84	Butyrophenone	495-40-9	1805	S,MS,RI	—	—	—	—	—	0.158
	Subtotal				6.903	4.782	5.090	8.151	8.788	4.000
Nitrogenous compounds										
85	2-Methylpyrazine	109-08-0	1268	S,MS,RI	0.170	—	—	0.274	0.298	0.321
86	2,5-Dimethylpyrazine	123-32-0	1324	S,MS,RI	—	—	—	0.054	0.157	0.186
87	2,6-Dimethylpyrazine	108-50-9	1330	S,MS,RI	0.216	—	—	0.374	0.330	0.626
88	2,3-Dimethylpyrazine	5910-89-4	1349	S,MS,RI	—	—	—	0.262	0.128	0.186
89	2-Ethyl-6-methylpyrazine	13925-03-6	1386	S,MS,RI	—	—	—	0.399	0.244	0.411
90	2,3,5-Trimethylpyrazine	14667-55-1	1405	S,MS,RI	0.097	0.029	0.070	1.440	0.905	1.542
91	2-Ethyl-3,5-dimethylpyrazine	13925-07-0	1463	S,MS,RI	—	—	—	0.663	0.203	0.617
92	Tetramethylpyrazine	1124-11-4	1476	S,MS,RI	0.262	—	—	1.710	0.544	2.068
93	2-Ethenyl-6-methylpyrazine	13925-09-2	1494	MS,RI	—	—	—	0.403	0.490	0.533
94	2-Ethyl-3,5,6-trimethylpyrazine	17398-16-2	1514	MS,RI	—	—	—	0.122	0.073	0.116
95	2-Acetylpyrrole	1072-83-9	1966	S,MS,RI	0.138	—	—	0.030	0.086	0.139
96	1H-Pyrrole-2-carboxaldehyde	1003-29-8	2013	S,MS,RI	—	—	—	—	—	0.482
	Subtotal				0.884	0.029	0.070	5.729	3.457	7.228
Aldehydes										
97	Hexanal	66-25-1	1084	MS,RI	1.310	—	—	0.744	0.631	0.819
98	(*E*)-2-Heptenal	18829-55-5	1325	MS,RI	0.824	—	—	—	—	—
99	Nonanal	124-19-6	1396	S,MS,RI	0.628	0.034	0.264	0.206	1.042	0.245
100	(*E*)-2-Octenal	2548-87-0	1431	S,MS,RI	0.282	—	—	—	—	0.055
101	3-Furaldehyde	498-60-2	1474	S,MS,RI	—	—	—	—	0.095	0.118
102	Decanal	112-31-2	1501	MS,RI	1.140	—	—	—	—	0.143
103	Benzaldehyde	100-52-7	1533	S,MS,RI	1.025	0.470	0.215	2.004	1.259	2.053
104	(*E*)-2-Nonenal	18829-56-6	1539	S,MS,RI	0.683	—	0.082	—	—	0.158
105	Phenylacetaldehyde	122-78-1	1654	S,MS,RI	0.412	0.524	0.367	0.661	0.734	0.636
106	(*E*,*E*)-2,4-Nonadienal	5910-87-2	1709	S,MS,RI	0.229	—	—	—	—	—
107	3-Ethylbenzaldehyde	34246-54-3	1718	S,MS	0.059	0.012	0.033	0.023	0.021	0.024
108	(*E*,*E*)-2,4-Decadienal	25152-84-5	1818	S,MS,RI	5.750	0.054	0.080	0.290	—	0.147
109	5-Methyl-2-thiophenecarboxaldehyde	13679-70-4	1825	MS	—	—	—	0.054	0.016	0.011
110	2-Phenyl-2-butenal	4411-89-6	1931	S,MS,RI	0.037	0.155	0.413	0.179	0.369	0.457
	Subtotal				12.381	1.249	1.455	4.162	4.169	4.866
Furans										
111	2-Pentylfuran	3777-69-3	1229	S,MS,RI	—	—	—	—	—	0.060
112	5-Methyl furfural	620-02-0	1584	S,MS,RI	—	—	—	—	0.125	0.229
113	2-Acetyl-5-methylfuran	1193-79-9	1625	S,MS,RI	0.087	—	—	—	—	0.038
114	Furfuryl alcohol	98-00-0	1667	S,MS,RI	—	0.262	0.308	0.431	0.177	0.546
115	5-Methyl-2-furanmethanol	3857-25-8	1726	MS,RI	—	—	—	—	0.100	0.108
116	3-Methyl-2(5H)-furanone	22122-36-7	1732	S,MS,RI	—	—	—	—	0.170	0.200
117	3-Phenylfuran	13679-41-9	1861	MS,RI	—	—	0.028	0.090	0.066	0.031
118	Dibenzofuran	132-64-9	2277	S,MS,RI	3.286	0.531	0.794	1.425	0.661	0.710
	Subtotal				3.373	0.793	1.130	1.946	1.299	1.922
Terpenes										
119	Limonene	138-86-3	1180	MS,RI	0.504	—	—	—	—	—
120	*α*-Copaene	3856-25-5	1490	MS,RI	0.398	—	—	—	—	—
121	Linanool	78-70-6	1547	MS,RI	1.215	—	—	—	—	—
122	*β*-Caryophyllene	87-44-5	1598	S,MS,RI	17.526	0.049	0.103	0.996	0.908	0.237
123	2-Isopropyl-5-methylcyclohexanol	1490-04-6	1640	MS,RI	0.299	—	—	—	—	—
124	(*E*)-*β*-farnesene	18794-84-8	1667	MS,RI	0.746	—	—	—	—	—
125	Humulene	6753-98-6	1671	MS,RI	0.150	—	—	—	—	—
126	(*S*)-*β*-bisabolene	495-61-4	1727	MS,RI	0.230	—	0.009	—	—	—
127	Geranyl acetone	3796-70-1	1856	S,MS,RI	6.331	0.044	0.074	0.467	0.050	0.071
	Subtotal				27.399	0.092	0.186	1.463	0.957	0.308
Sulfur compounds										
128	Benzothiazole	95-16-9	1955	S,MS,RI	0.268	0.049	0.017	0.025	0.039	0.046
129	4,5-Dimethylthiazole	3581-91-7	1377	MS,RI	0.162	—	—	—	—	0.198
130	Dimethyl trisulfide	3658-80-8	1384	S,MS,RI	—	—	—	—	0.034	—
	Subtotal				0.430	0.049	0.017	0.025	0.073	0.244
Others										
131	Styrene	100-42-5	1259	S,MS,RI	0.219	0.254	0.690	1.039	0.532	0.790
132	1,2-Dimethoxybenzene	91-16-7	1733	MS,RI	0.083	0.018	—	—	—	—
133	1,4-Dimethoxybenzene	150-78-7	1747	S,MS,RI	—	—	—	—	—	0.026
134	Naphthalene	91-20-3	1750	S,MS,RI	0.494	0.066	0.107	0.221	0.143	0.182
135	1,3-Dimethoxybenzene	151-10-0	1757	S,MS,RI	0.145	0.066	0.123	0.020	0.093	0.074
136	1-Methylnaphthalene	90-12-0	1861	S,MS,RI	0.184	0.012	0.073	—	0.100	0.069
137	3-Ethyl-2-methyl-1,3-hexadiene	61142-36-7	1418	MS	1.717	—	—	—	—	—
138	Pentadecane	629-62-9	1500	MS,RI	—	0.045	0.090	0.160	0.126	0.162
139	Hexadecane	544-76-3	1600	S,MS,RI	—	0.081	0.153	0.294	0.196	0.224
	Subtotal				2.841	0.542	1.236	1.734	1.191	1.526

^a^: NIST 11 was used to qualitative analysis, with matching degree ≥ 80; RI: retention index; S: standard substance was used to qualitative analysis; ^b^: The area normalization method was used to express the relative content of the compounds. MS: compounds identified by MS spectra.

**Table 2:** Analysis of active aroma compounds in Daqu after storage (S-3M).

No.	Compounds	CAS Number	Aroma Description	Identification ^a^	RI		Olfactory Intensity	
					DB-WAX ^b^	HP-5 ^c^	DB-WAX	HP-5
1	Ethyl 3-methylbutanoate	108-64-5	fruity	MS, aroma, RI, S	1071	859	2.2	2.0
2	Ethyl butyrate	105-54-4	fruity	MS, aroma, RI, S	1026	—	1.3	—
3	Ethyl valerate	539-82-2	fruity	MS, aroma, RI, S	1142	898	1.0	3.5
4	Limonene	138-86-3	sour/sweat	MS, aroma, RI, S	1189	—	2.7	—
5	Ethyl hexanoate	123-66-0	fruity	MS, aroma, RI, S	1240	1001	2.5	3.0
6	2,6-Dimethylpyrazine	108-50-9	wheat/roasted	MS, aroma, RI, S	1347	—	4.0	—
7	2,3-Dimethylpyrazine	5910-89-4	wheat/cocoa	MS, aroma, RI, S	1349	922	—	3.7
8	Dimethyl trisulfide	3658-80-8	garlic	MS, aroma, RI, S	1370	975	4.0	3.8
9	3-Ethyl-2-methylpyrazine	15707-23-0	roasted	MS, aroma, RI, S	1395	—	1.0	—
10	Nonanal	124-19-6	oily	MS, aroma, RI, S	1396	1108	—	3.0
11	2,3,5-Trimethylpyrazine	14667-55-1	earthy	MS, aroma, RI, S	1410	—	3.3	—
12	2-Octanol	123-96-6	soapy/creamy	MS, aroma, RI, S	1412	—	3.5	—
13	(*E*)-2-Octenal	2548-87-0	grassy/fatty	MS, aroma, RI, S	1429	—	2.5	—
14	Acetic acid	64-19-7	acetic	MS, aroma, RI, S	1452	—	4.0	—
15	1-Heptanol	111-70-6	grassy	MS, aroma, RI, S	1452	980	—	2.5
16	2-Ethyl-3,5-dimethylpyrazine	13925-07-0	boiled potato	MS, aroma, RI, S	1464	1085	4.0	2.5
17			herbal	aroma	1480	—	1.0	—
18	2-Ethylhexanol	104-76-7	grassy	MS, aroma, RI, S	1489	—	1.0	—
19	Benzaldehyde	100-52-7	bitter almond	MS, aroma, RI, S	1508	961	3.7	2.0
20	(*E*)-2-Nonenal	18829-56-6	grassy/fatty	MS, aroma, RI, S	1536	—	2.5	—
21			minty	aroma	1574	—	2.5	—
22	*β*-Caryophyllene	87-44-5	woody	MS, aroma, RI, S	1585	—	3.8	—
23			wheat	aroma	1602	—	2.5	—
24	Butanoic acid	107-92-6	sour and stinky	MS, aroma, RI, S	1628	—	4.0	—
25	*γ*-Butyrolactone	96-48-0	wheat	MS, aroma, RI, S	1643	—	2.3	—
26	Phenylacetaldehyde	122-78-1	penicillin/grassy	MS, aroma, RI, S	1654	1044	—	3.0
27	Furfuryl alcohol	98-00-0	burnt/caramel	MS, aroma, RI, S	1666	—	1.7	—
28	Ethyl benzoate	93-89-0	musty	MS, aroma, RI, S	1672	1173	2.0	2.5
29	3-Methylbutanoic acid	503-74-2	sour and stinky	MS, aroma, RI, S	1680	—	4.0	—
30	Diethyl succinate	123-25-1	wheat/fruity	MS, aroma, RI, S	1704	—	1.0	—
31	Naphthalene	91-20-3	grassy	MS, aroma, RI, S	1750	1182	—	3.8
32			herbal(powdery)	aroma	1760	—	3.8	—
33	Ethyl phenylacetate	101-97-3	sweety	MS, aroma, RI, S	1793	1247	—	2.0
34	2,4-Decadienal	25152-84-5	fatty/grassy	MS, aroma, RI, S	1818	1316	2.0	2.2
35	1-Phenylethanol	98-85-1	vanilla	MS, aroma, RI, S	1820	—	1.5	—
36			wheat	aroma	1832	—	2.0	—
37	Geranyl acetone	3796-70-1	grassy	MS, aroma, RI, S	1856	1453	—	2.5
38			bitter	aroma	1857	1349	3.3	3.2
39			sweet	aroma	1862	1390	3.0	3.5
40	Hexanoic acid	142-62-1	sour	MS, aroma, RI, S	1866	—	3.2	—
41	Guaiacol	90-05-1	woody	MS, aroma, RI, S	1871	1096	4.0	3.0
42	Benzyl alcohol	100-51-6	sweet/floral	MS, aroma, RI, S	1877	—	2.5	—
43	Phenethyl alcohol	60-12-8	honey	MS, aroma, RI, S	1901	1114	4.0	4.0
44	2-Phenyl-2-butenal	4411-89-6	sweet	MS, aroma, RI, S	1917	—	3.1	—
45	4-Ethyl-2-methoxyphenol	2785-89-9	clove	MS, aroma, RI, S	2018	1279	4.0	3.5
46	*γ*- Nonanolactone	104-61-0	coconut	MS, aroma, RI, S	2020	1368	4.0	2.8
47	*p*-Cresol	106-44-5	animal	MS, aroma, RI, S	2078	—	3.0	—
48	2-Methoxy-4-vinylphenol	7786-61-0	bake/bitter	MS, aroma, RI, S	2184	1309	3.2	1.2
49	Methyl palmitate	112-39-0	wax	MS, aroma, RI, S	2218	1929	4.0	3.9
50	Hexanal	66-25-1	fatty/grassy	MS, aroma, RI, S	1078	838	3.1	2.0

^a^: NIST 11 was used to qualitative analysis, with matching degree ≥ 80; RI: retention index; S: standard substance was used to qualitative analysis; ^b^: Linear retention index of DB-wax; ^c^: Linear retention index of HP-5.

**Table 3:** OAV calculation for the quantitative compounds.

No.	Compounds	Threshold ^a^/(ppb)	OAVs
6	2,6-Dimethylpyrazine	200	2
7	2,3-Dimethylpyrazine	10823.7	<1
9	3-Ethyl-2-methylpyrazine	130	<1
10	Nonanal	1	655
11	2,3,5-Trimethylpyrazine	10	24
13	(*E*)-2-Octenal	3	40
16	2-Ethyl-3,5-dimethylpyrazine	1	43
19	Benzaldehyde	30	5
20	(*E*)-2-Nonenal	0.6	339
22	*β*-Caryophyllene	64	2
25	*γ*- Butyrolactone	50	11
26	Phenylacetaldehyde	4	447
27	Furfuryl alcohol	100	28
34	2,4-Decadienal	0.07	2746
37	Geranyl acetone	60	1
41	Guaiacol	5.5	172
44	2-Phenyl-2-butenal	-	-
45	4-Ethyl-2-methoxyphenol	6.9	40
46	*γ*- Nonanolactone	21	10
48	2-Methoxy-4-vinylphenol	3	122
50	Hexanal	25.48	81

^a^: Check the threshold of the corresponding compound on http://www.leffingwell.com (accessed on 23 July 2020).

## Data Availability

Date of the compounds are available from the authors.

## Supplementary Materials

The following supporting information can be downloaded at: https://www.mdpi.com/article/10.3390/foods11010116/s1. Figure S1 Baijiu flavour wheel [5]; Figure S2 TIC diagram of *Daqu* after storage (S-3M); Table S1 Sample code and status.
