# Supplementary material for: HS-SPME Combined with GC-MS/O to Analyze the Flavor of Strong Aroma Baijiu Daqu
Source: Foods. 2022 Jan 3;11(1):116. doi: 10.3390/foods11010116 (PMC8750912; doi:10.3390/foods11010116)
Supplement: Supplementary file 1 [file foods-11-00116-s001.zip › foods-1493214-supplementary.pdf]

Table S1 Samples code and status.

Sample name	Different states of *Daqu*
S-0D	*Daqu* blank-pressed (0 Day)
S-4D	*Daqu* after early fermentation (4 Day)
S-6D	*Daqu* with low temperature culture (6 Day)
S-10D	*Daqu* with slow rising stage (10 Day)
S-22D	*Daqu* with mature stage (22 Day)
S-3M	*Daqu* after storage (3 Month)

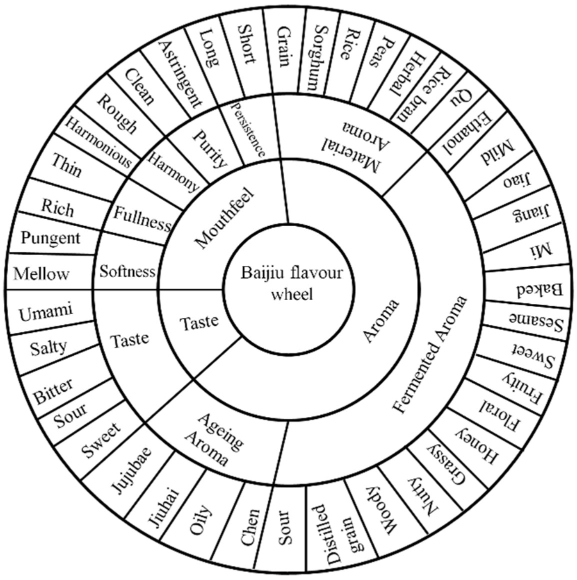

Figure S1 Baijiu flavour wheel

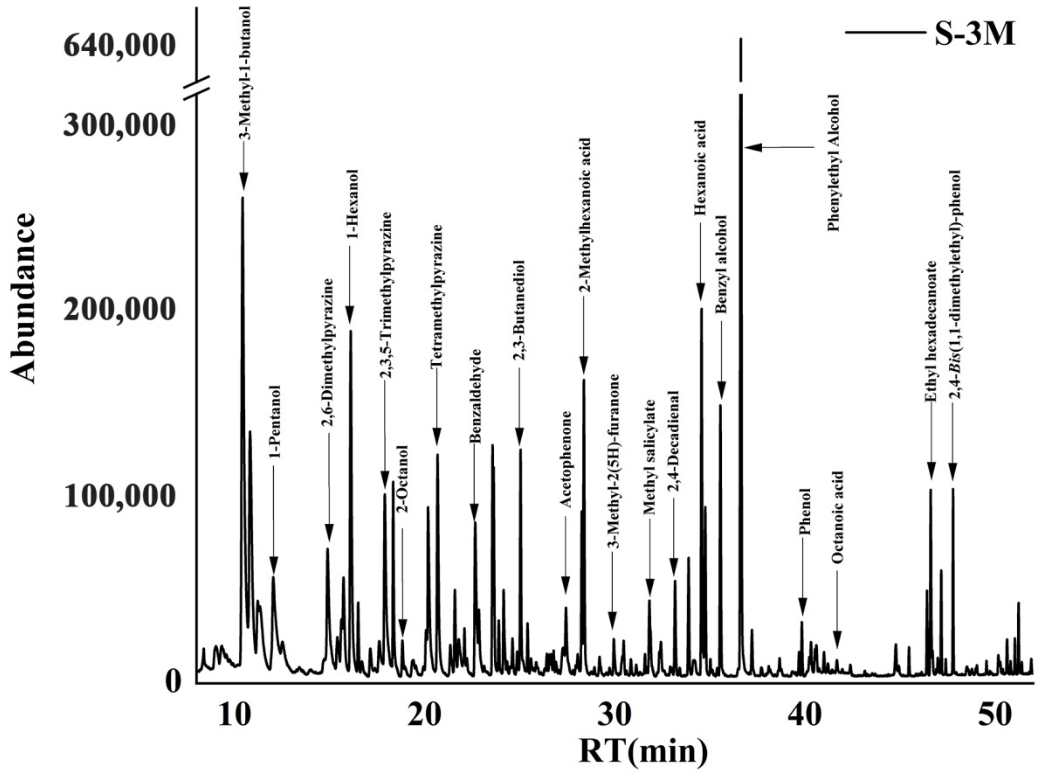

Figure S2 TIC diagram of Daqu after storage (S-3M)
